# Supplementary material for: CryptoBank: A resource for the identification and prediction of cryptic sites in proteins
Source: Sci Adv. 2026 Apr 22;12(17):eady6364. doi: 10.1126/sciadv.ady6364 (PMC13267282; doi:10.1126/sciadv.ady6364)
Supplement: Supplementary file 1 — Figs. S1 to S9 Table S1 [file sciadv.ady6364_sm.pdf]

Science Advances
AAAS

Supplementary Materials for

CryptoBank: A resource for the identification and prediction of cryptic sites in proteins

Pedro Febrer Martinez *et al.*

Corresponding author: Francesco L. Gervasio, francesco.gervasio@unige.ch

Sci. Adv. **12**, eady6364 (2026)
DOI: 10.1126/sciadv.ady6364

This PDF file includes:

Figs. S1 to S9
Table S1

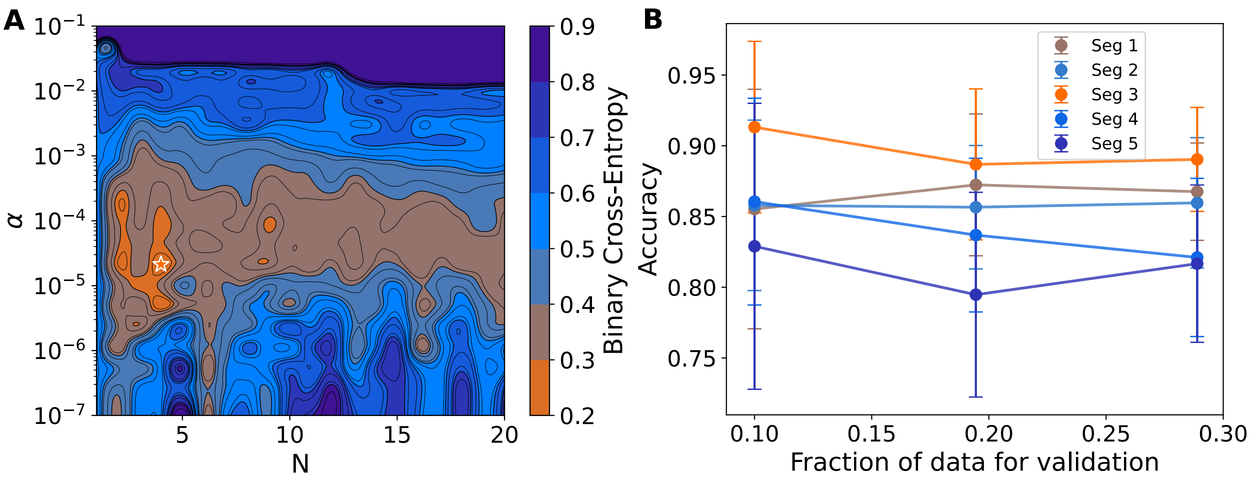

Figure S1: **Classifier training**. Hyperparameter scan over the number of shells, N , regularization strength, α. and ligand segments, S. (**A**) 2D landscape of binary cross-entropy loss for the classifier after training, evaluated on the validation set. The star marks the optimal combination of N and α. (**B**) Validation accuracy as a function of the fraction of data left out for validation. The analysis compares classifiers trained with their respective optimal N and α values, further examining performance across varying numbers S.

Outliers Identification

To investigate cases where individual ligands exhibit crypticity scores that deviate substantially from the average crypticity of the binding site, we computed a ligand-site deviation score for each ligand:

$$\Delta_i = \text{lig\_mean\_score}_i - \text{site\_mean\_score}_i$$

where lig_mean_score_i is the average crypticity score of ligand i within site i, and site_mean_score_i is the average crypticity score of the site it binds. We limited this analysis to binding sites with more than one unique ligand, as such cases may highlight how ligands of different size and orientation can differentially expose cryptic and non-cryptic regions. To systematically detect extreme deviations, we normalized the deviation scores by calculating z-scores:

$$z_i = \frac{\Delta_i - \mu}{\sigma}$$

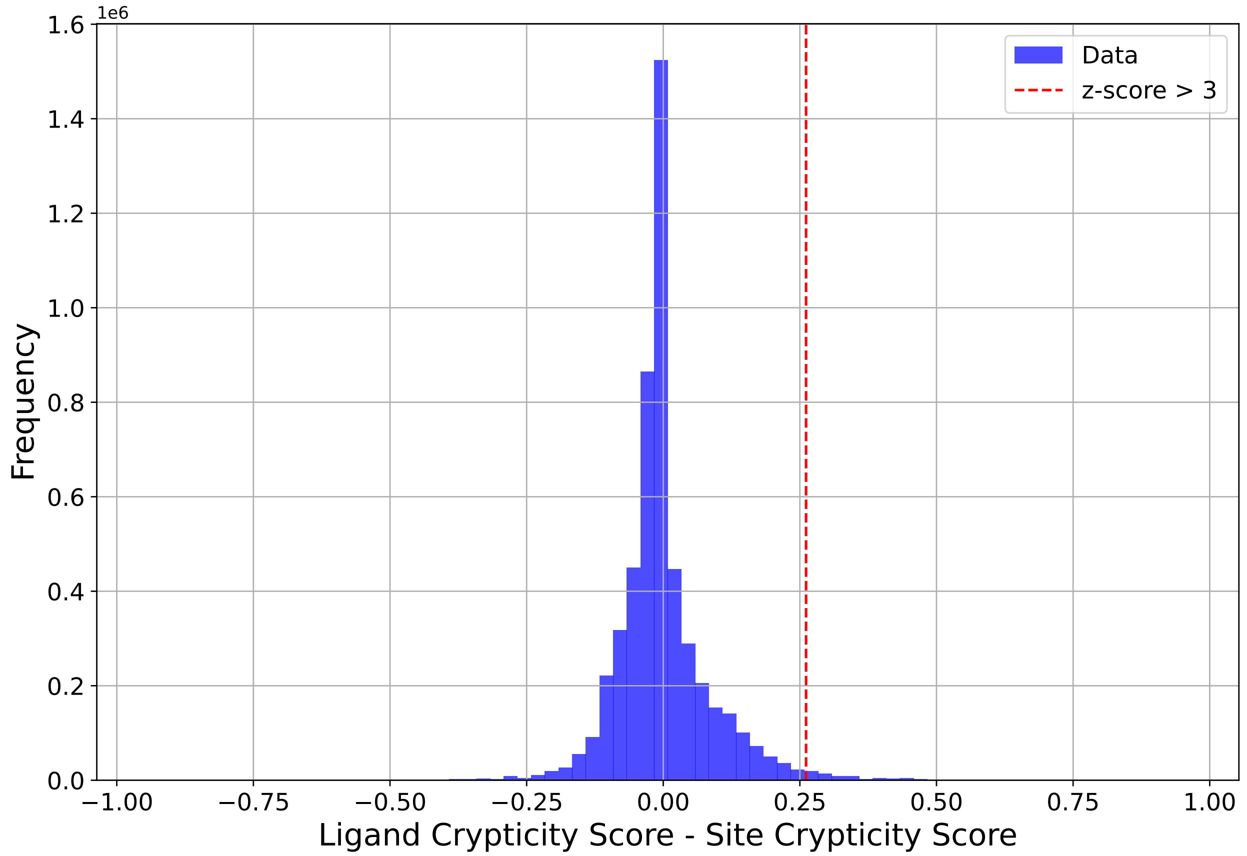

Figure S2: **Outliers Identification**. Histogram showing the distribution of the difference between the ligand crypticity score and the crypticity score of the site it binds to. The vertical dashed line indicates the threshold for right-tail outliers, defined as having a z-score greater than 3 ($z_i > 3$).

where μ and σ are the mean and standard deviation of the Δ_i distribution, respectively. Ligands with $|z_i| > 3$ were considered outliers.

Estimate of ligand-protein volume overlap in cryptic sites

We estimated the overlapping volume between ligand and protein atoms in the corresponding structurally aligned apo and holo structures using a Monte Carlo sampling approach. For each ligand, we first computed its total volume by summing the volumes of individual atoms, approximated as spheres with element-specific van der Waals radii. We then randomly sampled points within each ligand atom and determined whether each point fell within the van der Waals radius of any nearby protein atom. To reduce computational cost, only protein atoms within a cutoff distance (e.g.,

5 Å) from the ligand were considered. This procedure was applied separately to the apo and holo protein structures. The fraction of points lying inside protein atoms in the apo structure provides an estimate of the ligand-apo overlap, while the fraction inside the holo structure provides the ligand-holo overlap.

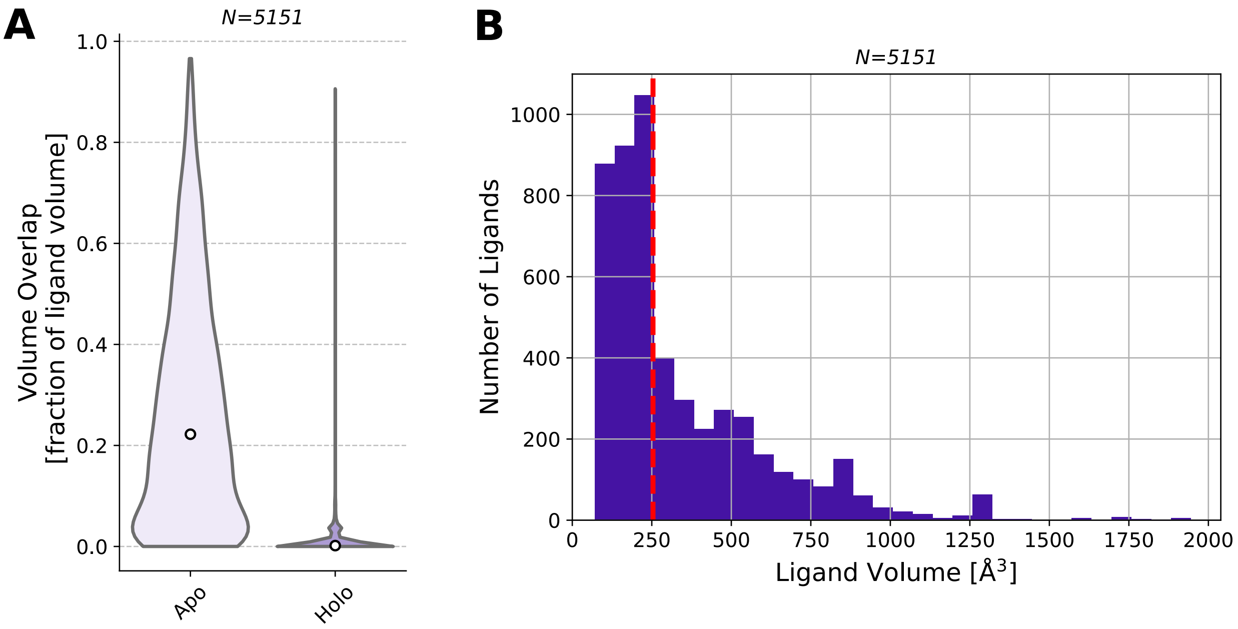

Figure S3: **Ligand-protein volume overlap and ligand volume.** (**A**) Violin plots showing the fraction of volume overlap between ligand–apo and ligand–holo atoms. Each plot corresponds to one representative apo–holo pair from the 5,151 identified cryptic sites, selected as the pair with the maximum crypticity score. (**B**) Histogram of the estimated ligand volumes for the ligands shown in panel *A*. For each apo–holo pair, the ligand is taken from the holo structure of the max-scoring pair. The red dashed line indicates the median volume, ≈ 250 Å^3.

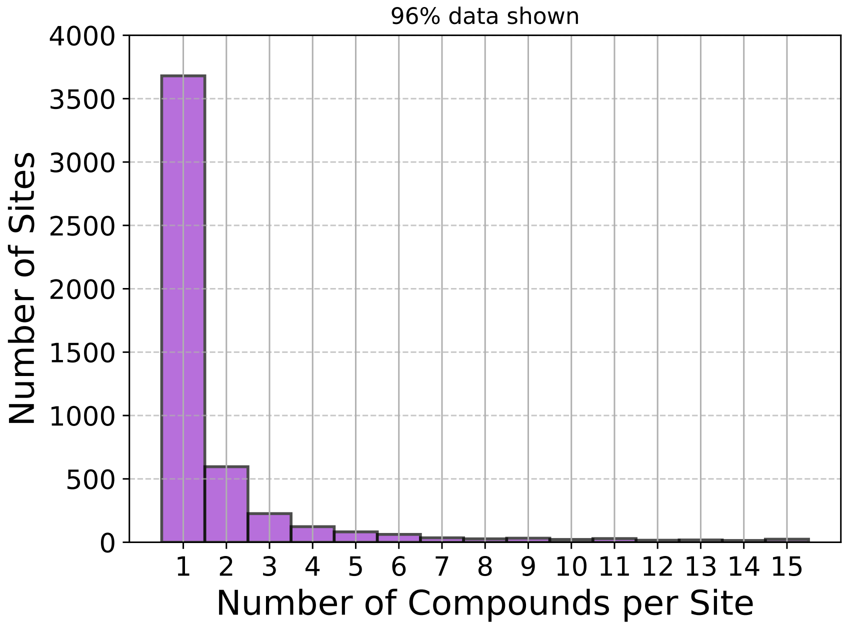

Figure S4: **Distribution of compounds bound per cryptic site.** Histogram showing the distribution of the number of chemically distinct compounds binding to each cryptic site. The count is capped at 15 compounds per site, with sites binding up to 15 compounds cumulatively representing 96% of the total sites. Overall, 28.6% of cryptic sites bind more than one compound.

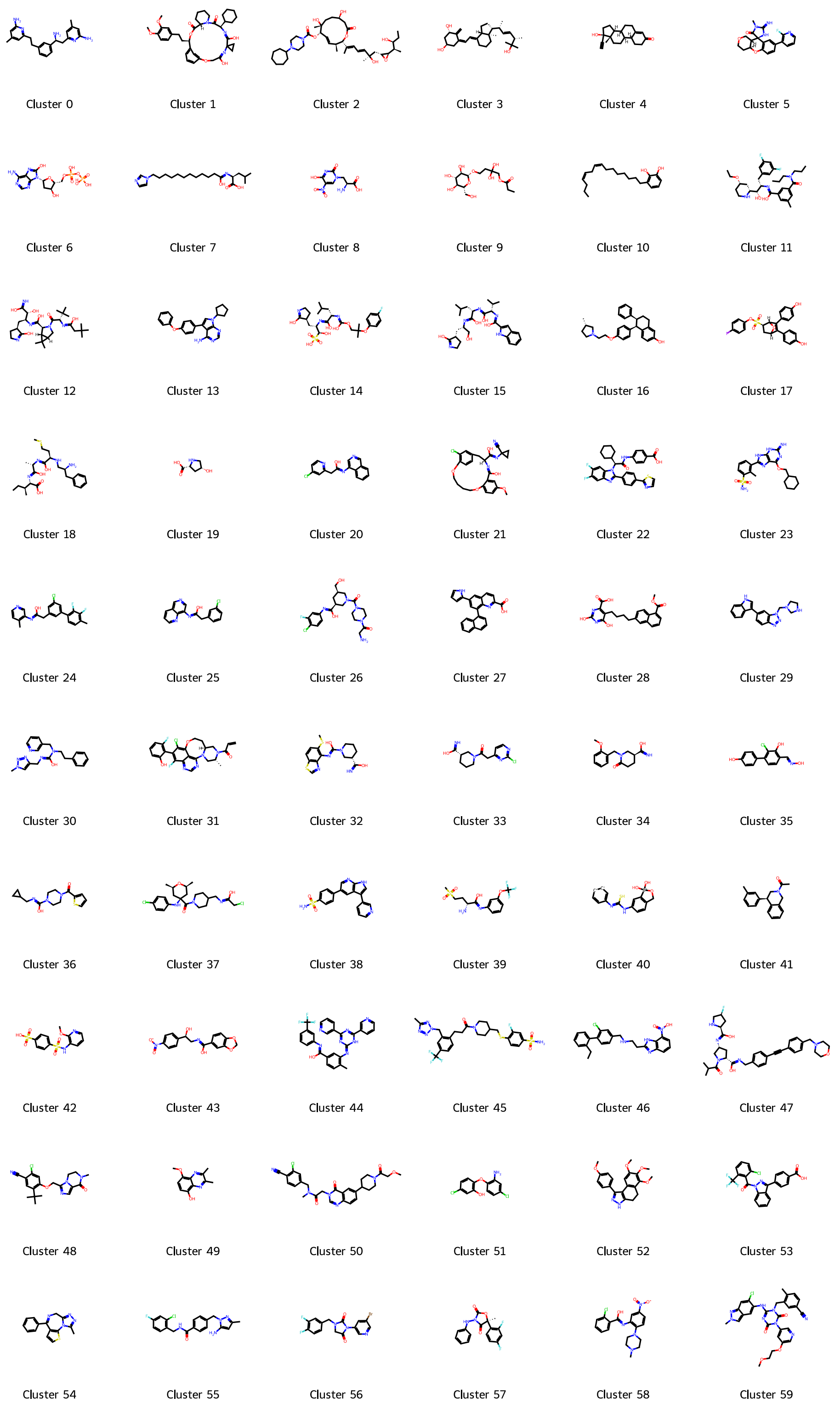

Figure S5: **Cluster representatives.** Chemical structures of the ligands selected as representatives for each cluster. The corresponding clusters are shown in Fig. S6A.

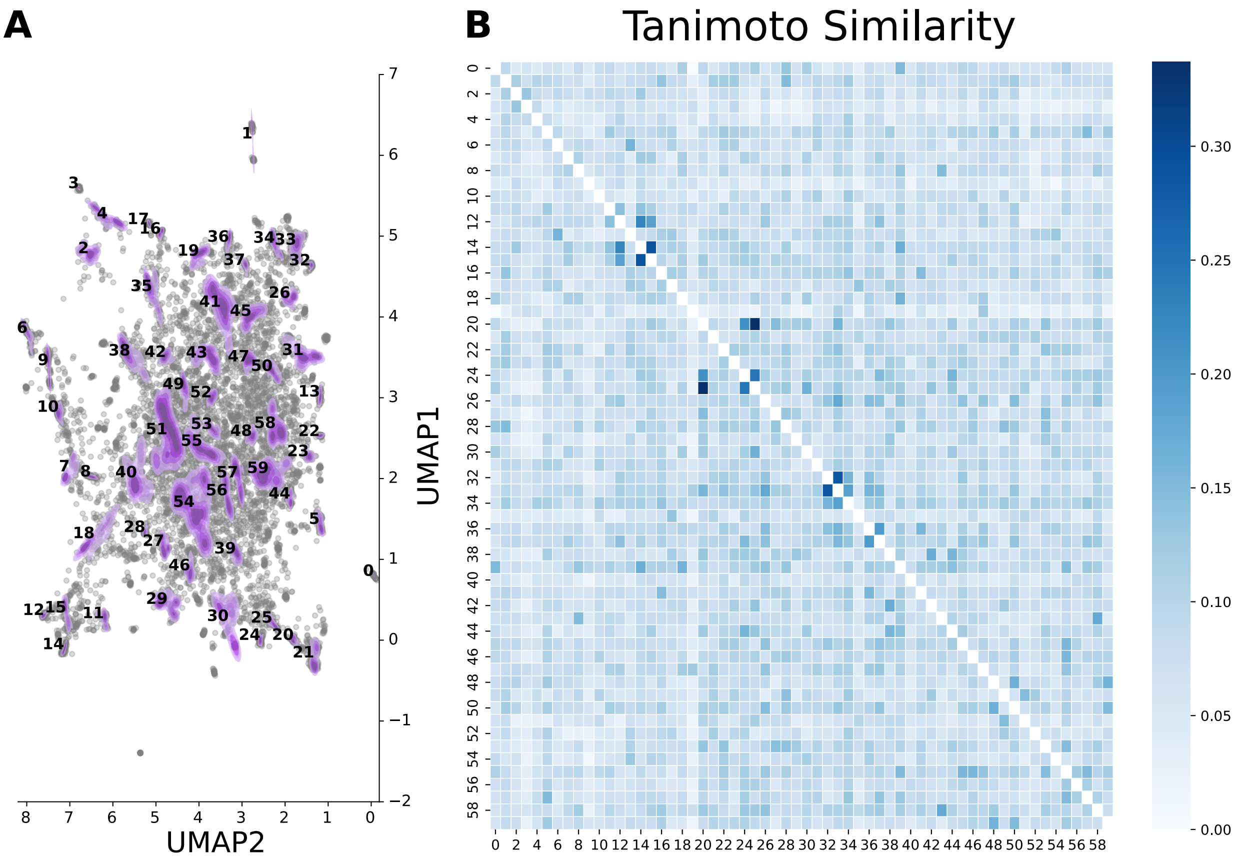

Figure S6: **Ligand clusters and similarity in chemical space.** (**A**) Chemical space of compounds found exclusively in cryptic binding sites, visualized using UMAP. Each point represents a compound, with clusters shown in purple and labelled numerically. Clustering was performed using HDBSCAN. Only compounds with a molecular weight below 1200 Da were included in the analysis. (**B**) Heatmap showing the pairwise Tanimoto similarity between cluster centroids. The colour bar indicates the degree of chemical similarity.

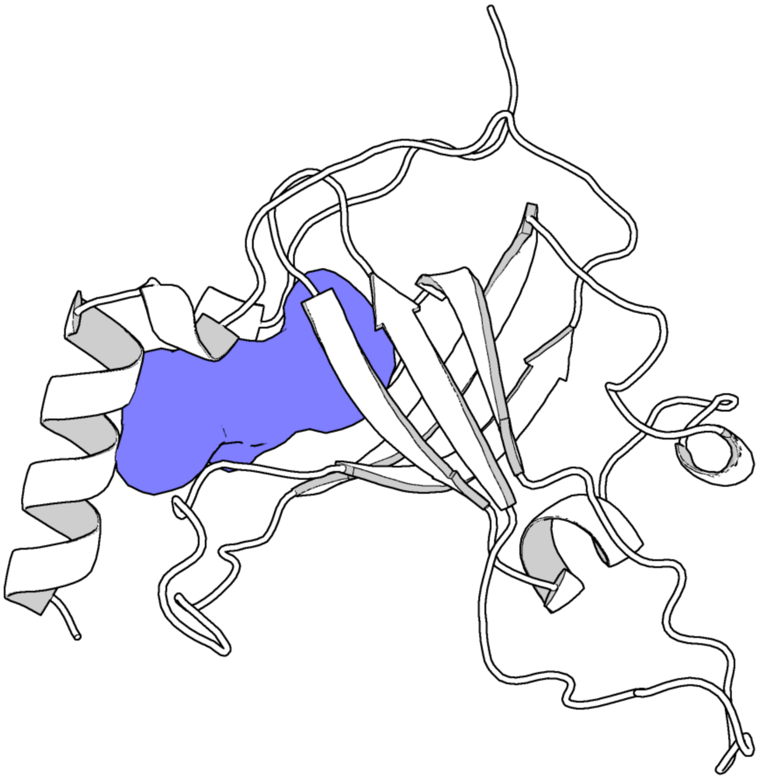

Figure S7: **TPP1 crystal pocket.** Structural representation of TPP1 shown as a white cartoon. The internal cavity identified in the cryo-EM structure (PDB: 7TRE) is displayed as a purple surface.

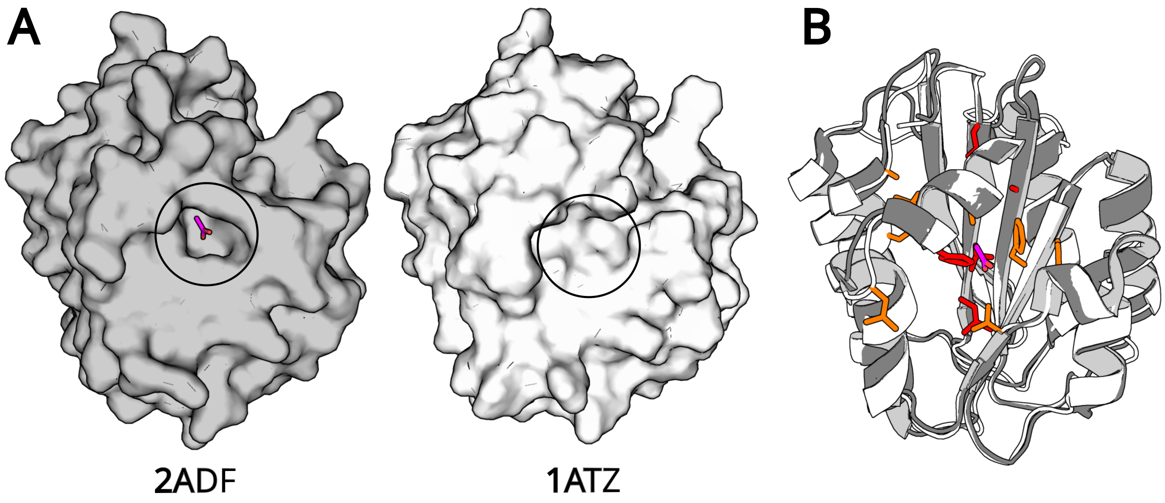

Figure S8: **VWF's A3 cryptic pocket.** (**A**) Exposure of the cryptic cavity by an acetic acid molecule in PDB ID 2ADF. The pocket is highlighted with a black circle. The corresponding region in PDB ID 1ATZ shows no cavity. Proteins are rendered as surfaces, with acetic acid shown in licorice representation. (**B**) Structural alignment of 2ADF and 1ATZ. Residues from 1ATZ predicted to form the cryptic pocket are rendered as sticks and colored by normalized crypticity score (red for scores above 0.6; orange for scores between 0.4 and 0.6). 1ATZ is shown as a white cartoon, while 2ADF is shown as a gray cartoon.

PLM fine-tuning for binding site prediction

In Figure S9 we collected the evaluation of the fine-tuned Prot-T5-XL-UniRef50 model performance across training, validation, and test set. While in the main text we show how PLM fine-tuning performs on exclusively cryptic sytems, this section highlights the extrapolation capabilities of a fine-tuned PLM focusing on binding site prediction, which effectively included more proteins and ligands. The ROC curve for the training set yields a high AUC of 0.96, because of the high data imbalance with a positive class ratio of 0.07 making it easier for the model to achieve high AUC. We select the best model as the one that is maximizing the cross-entropy loss on the validation set yielding an AUC of 0.89. Since the validation data share a similar imbalance with a positive class ratio of 0.06, the ROC AUC might be overestimate the extrapolation behavior to systems not seen during training. Benchmarking against the test set results in an AUC of 0.81 for the ROC curve (compare Figure S9A), indicating that unique sequences impose a significant challenge for the PLM, which is in agreement with the result of the fine-tuning experiment in the main text. To highlight the model's ability to achieve high precision we compute the model's precision-recall curves shown in Figure S9B. According to this metric, the various datasets differ even more significantly in terms of their AUCs. The precision on the training data is high with a PR AUC of 0.80 corresponding to an increase in precision of an order of magnitude compared to a random model. Comparing to the precision of the selected model with respect to the validation set a significant decrease in PR AUC to 0.51 can be seen. Importantly the model retains significant precision indicating it is capable of extrapolation to new systems with a corresponding 9-fold increase in precision compared to random predictions. Benchmarking against the test set yielded an AUC 0.32 for the precision-recall curve. While the high accuracy obtained on the test set can be misleading for imbalanced data the PR AUC instead reveals the significant drop in performance. The retained precision corresponds to a 5-fold increase in precision compared to a random model.

The results of this second fine-tuning experiment indicate that signal about crypticity is encoded at the sequence level and that non-cryptic binding sites can be inferred from learning about cryptic ones. Furthermore, the model demonstrates a limited but notable ability to extrapolate signals to unique sequences, emphasizing its generalization capability beyond the training data.

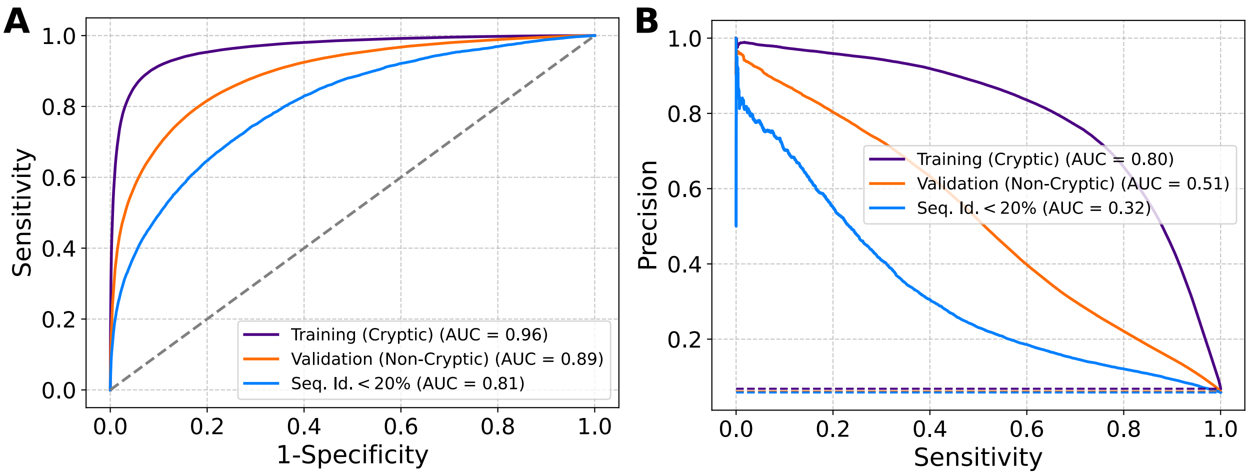

Figure S9: **Performance of the 'Prot-T5-XL-UniRef50' model in predicting binding sites after fine-tuning evaluated on training, validation and test set.** (**A**) ROC curves showing AUCs of 0.96, 0.89 and 0.81 respectively, while random prediction will lead to an AUC of 0.5. (**B**) Precision-recall curves show AUCs of 0.80, 0.51 and 0.32 respectively, demonstrating high precision for the imposed imbalanced binary classification task. Performance of random predictions corresponds to the fraction of the positive class and is maximally at 0.07 for all sets visualized as dashed lines with matching colors.

Table S1: **SWISH-X and unbiased simulation parameters.**

Protein	PDB ID	Simulation type	Replicas	Temperature	Force Field	λ window	Opes Explore pace*
HyBcl-2-4	6WH0†	SWISH-X	6×250 ns	300–350 K	DES-Amber	1–1.35	500
		unbiased	1×750 ns	300 K	–		
TPP1	7TRE†	SWISH-X	6×250 ns	300–330 K	AMBER99-ILDN	1–1.35	5000
		unbiased	1×750 ns	300 K	–		
VWF A3	1ATZ†	SWISH-X	6×250 ns	300–350 K	DES-Amber	1–1.35	500
		unbiased	1×750 ns	300 K	–		
ROR2 CRD	9FSE†	SWISH-X	6×250 ns	300–350 K	DES-Amber	1–1.35	500
		unbiased	1×750 ns	300 K	–		

† = apo structure

* = given as number of simulation steps ($\Delta t = 2$ fs)
